# Spiral imaging with off-resonance reconstruction for MRI-guided cardiovascular catheterizations using commercial off-the-shelf nitinol guidewires

**DOI:** 10.1186/1532-429X-18-S1-P216

**Published:** 2016-01-27

**Authors:** Adrienne E Campbell-Washburn, Toby Rogers, Kanishka Ratnayaka, Burcu Basar, Ozgur Kocaturk, Hui Xue, Robert J Lederman, Michael S Hansen, Anthony Z Faranesh

**Affiliations:** 1 Cardiovascular and Pulmonary Branch, Division of Intramural Research, National Heart, Lung, and Blood Institute, National Institutes of Health, Bethesda, MD USA; 2 Department of Cardiology, Children's National Medical Center, Washington, DC USA; 3 Institute of Biomedical Engineering, Bogazici University, Istanbul, Turkey USA

## Background

MRI-guidance of cardiovascular catheterization offers improved soft-tissue contrast and reduced ionizing radiation exposure. The application of MRI-guidance to complex catheterization procedures has been limited by the unavailability of guidewires that are safe and visible under MRI. Here, we use RF-efficient spiral imaging for MR-guided cardiovascular catheterization, with real-time off-resonance reconstruction for improved visualization of off-the-shelf nitinol guidewires.

## Methods

MRI-guided left and right heart catheterizations were performed on a swine using a commercial nitinol guidewire (0.035"/145 cm Nitrex, Covidien, Plymouth, MN) and balloon-tipped catheter (7 Fr, Arrow-Teleflex, Limerick, PA) with spiral imaging (gradient echo, 16 interleaves, TE/TR =0.86/11 ms, flip = 10°, FOV = 300 mm x 300 mm, matrix = 192 x 192, slice thickness = 6 mm). To enhance guidewire visualization, we exploited the off-resonance signal near the guidewire. Using a custom reconstruction framework (Gadgetron [1]), the imaging data was reconstructed at two different off-resonance frequencies (± 100 Hz) and the images were subtracted to produce guidewire-enhanced images. The method was implemented such that operators could rapidly toggle between anatomical imaging, saturation pre-pulses for visualization of gadolinium-filled balloon [2] and guidewire-enhanced imaging, as-needed throughout the procedure.

RF-induced heating of the guidewire/catheter configuration was evaluated in an ASTM 2182 phantom. A fiber-optic temperature probe (0.007" OpSens, Quebec, Canada) affixed to the guidewire tip measured temperature during 2 minutes of continuous scanning with the spiral sequence and our standard real-time imaging sequence (Cartesian bSSFP, TE/TR = 1.31/2.62 ms, flip angle = 45°).

## Results

**Figure 1:**
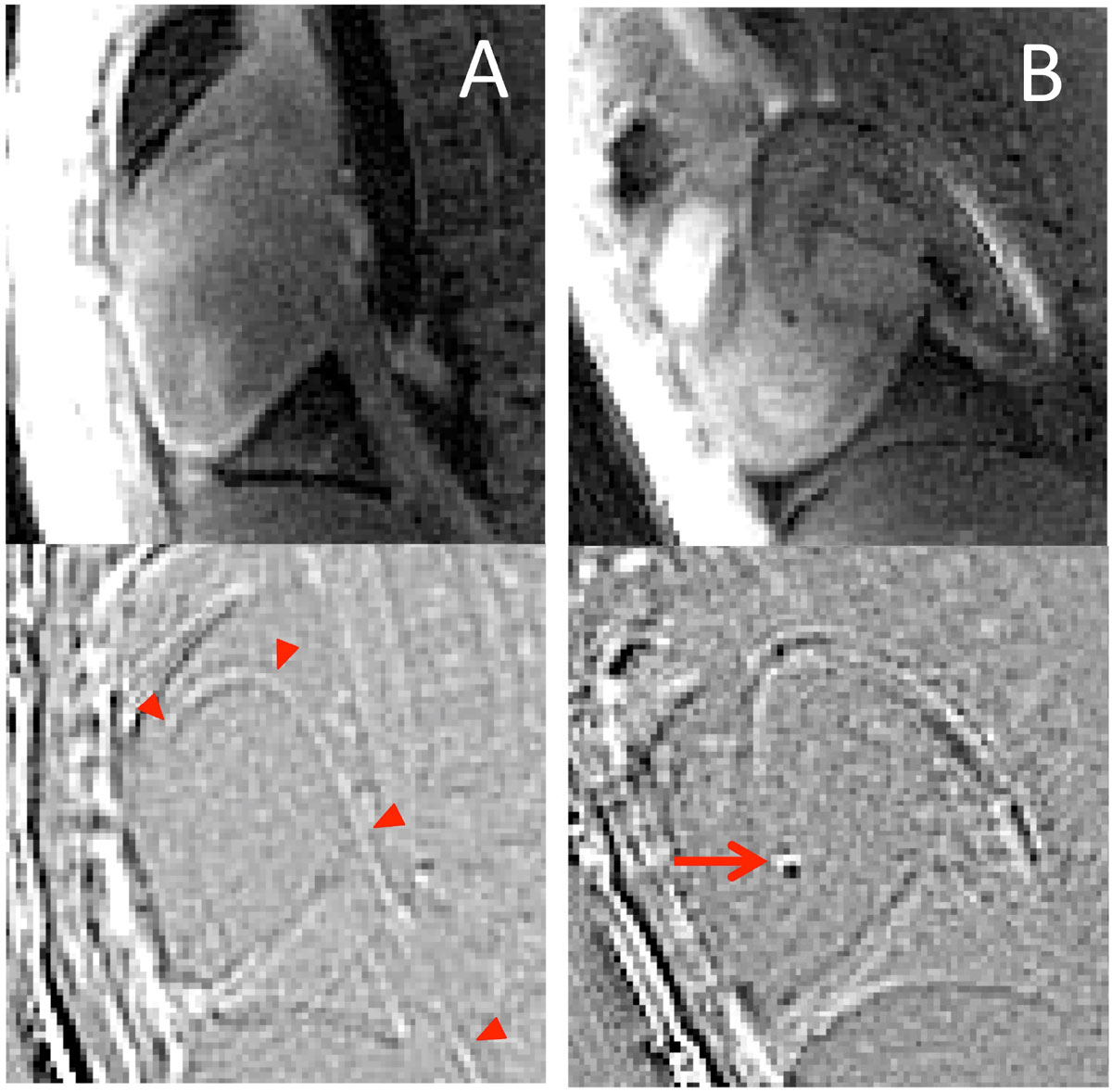
**Images from right heart (A) and left heart (B) catheterizations, comparing standard anatomical imaging (top) to guidewire-enhanced images (bottom)**. Improved guidewire visualization compared to signal-void imaging (A, red arrowheads) and a unique in-plane guidewire tip signal (B, red arrow) are demonstrated. Tissue boundaries are also visible in the guidewire-enhanced images which provides anatomical context for navigation.

The spiral sequence generated 6 frames/s. Guidewire-enhanced images offered improved delineation of the guidewire shaft, compared to standard signal void visualization (Figure 1A), and a unique guidewire tip artifact when in-plane (Figure 1B). These images also preserve tissue boundaries, which is valuable to provide anatomical context for guidewire navigation.

**Figure 2:**
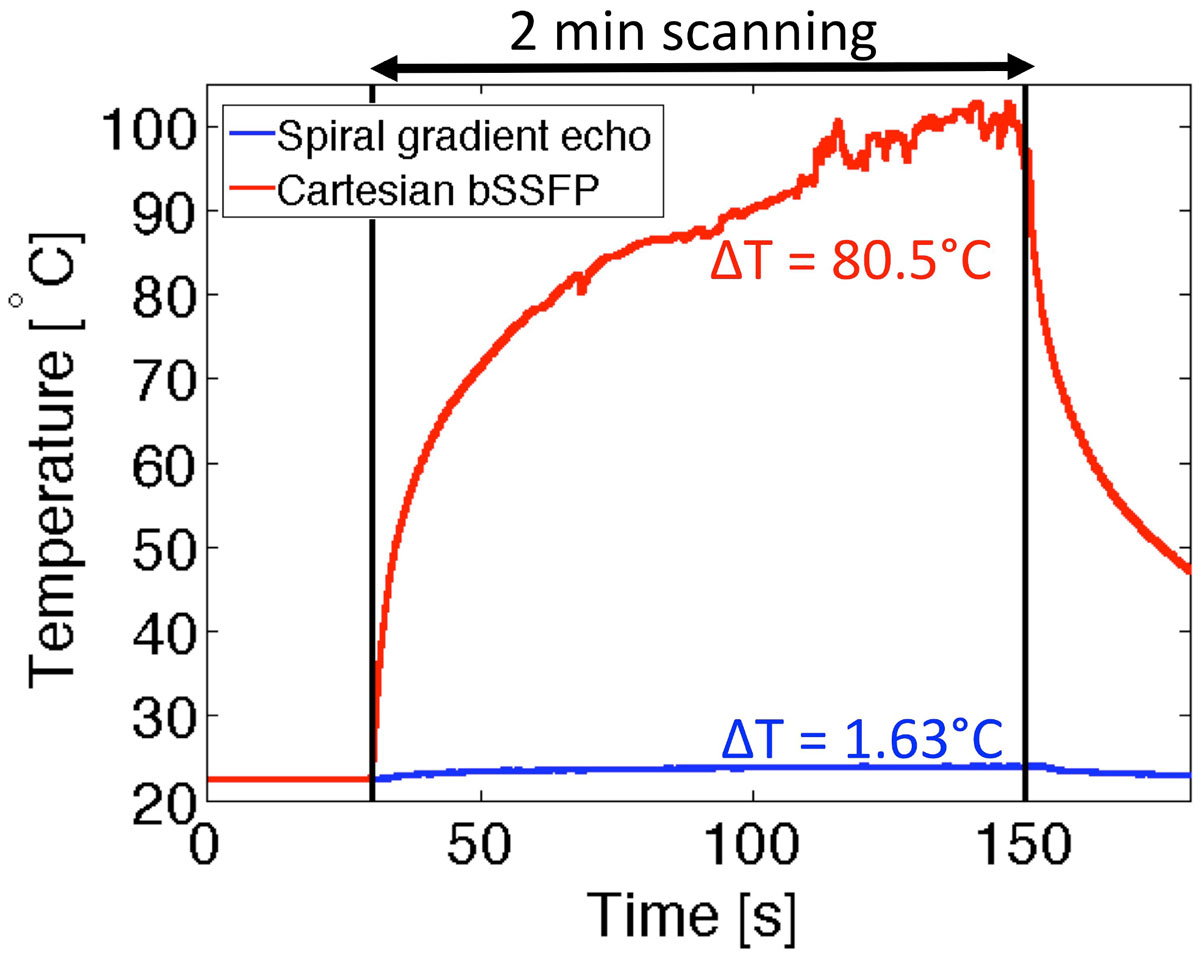
**Temperature at the tip of the nitinol guidewire measured during 2 minutes of continuous scanning using Cartesian bSSFP (TE/TR = 1.31/2.62 ms, flip angle = 45°) (A) and spiral gradient echo (TE/TR = 0.86/8.16 ms, flip angle = 10°) (B)**. Signal oscillation is observed in the non-linear range of the temperature probe (>85°C).

Substantial heating (ΔT = 80.5°C) was observed using our standard real-time Cartesian bSSFP sequence. Heating was reduced to below allowable limits using spiral gradient echo imaging (ΔT = 1.63°C) (Figure 2).

## Conclusions

This visualization method is particularly flexible because it uses a targeted reconstruction of standard anatomical imaging data. This method may enable safe MRI-guided cardiovascular catheterizations using commercially available nitinol guidewires.
